# Supplementary material for: Primary Reason for Drinking Among Current, Former, and Never Flushing College Students
Source: Int J Environ Res Public Health. 2019 Jan 13;16(2):211. doi: 10.3390/ijerph16020211 (PMC6352046; doi:10.3390/ijerph16020211)
Supplement: Supplementary file 1 [file ijerph-16-00211-s001.pdf]

Table S1. Stratification of a nationally representative sample of college students in South Korea.

	4-year Courses of Study		2-year Courses of Study		Total
	**Population ratio (%)**	**Students(colleges)**	**Population ratio (%)**	**Students(colleges)**	**Students(colleges)**
Seoul	12.6	630 (10)	4.2	210 (3)	840 (13)
Incheon/Gyeonggi	9.4	471 (8)	8.1	403 (7)	874 (15)
Gangwon	5.6	280 (5)	2.5	124 (2)	404 (7)
Daejeon/Chungjeong	11	552 (9)	4.7	233 (4)	785 (13)
Gwangju/Jeolla	8.5	426 (7)	4.9	245 (4)	671 (11)
Daegu/Gyeongbuk	8.3	417 (7)	5.4	270 (4)	687 (11)
Busan/Ulsan/Gyeongnam	9.8	488 (8)	5	251 (4)	739 (12)
Total	65.3	3264 (54)	34.7	1736 (28)	5000 (82)

Table S2. Results of the logistic regression analysis performed to investigate the association between factors and a one-unit increase in the Alcohol Use Disorders Identification Test (AUDIT).

	AUDIT Scores											
	Current Flusher				Former Flusher				Never Flusher			
	**OR**	**95% CI**			**OR**	**95% CI**			**OR**	**95% CI**		
**Primary Reason for Drinking**												
Peer Pressure	1.00	-			1.00	-			1.00			
Pleasure	0.32	(0.25	-	0.40)	0.63	(0.25	-	1.56)	0.35	(0.30	-	0.41)
Stress/Depression	0.34	(0.27	-	0.44)	0.88	(0.40	-	1.96)	0.50	(0.41	-	0.60)
Boredom	0.76	(0.57	-	1.01)	0.39	(0.15	-	1.07)	0.74	(0.59	-	0.93)
Other	1.80	(0.83	-	3.89)	-				1.29	(0.75	-	2.24)
**Sex**												
Male	1.00	-			1.00	-			1.00			
Female	1.09	(0.90	-	1.32)	0.84	(0.41	-	1.70)	0.87	(0.75	-	1.00)
**Year Level**												
1	1.00	-			1.00	-			1.00			
2	0.80	(0.64	-	1.00)	1.79	(0.85	-	3.80)	0.97	(0.83	-	1.14)
3	0.93	(0.71	-	1.21)	2.49	(0.98	-	6.33)	1.02	(0.83	-	1.24)
≥4	1.45	(1.12	-	1.87)	2.16	(0.84	-	5.56)	1.04	(0.86	-	1.26)
**Major**												
Humanities and Social Sciences	1.00	-			1.00	-			1.00			

Engineering / Natural Sciences	0.97	(0.80	-	1.17)	1.24	(0.67	-	2.29)	0.93	(0.81	-	1.07)
Liberal Arts	1.11	(0.83	-	1.48)	2.18	(0.79	-	6.04)	0.95	(0.77	-	1.16)
**Grade Point Average**												
≥4.0	1.00	-			1.00	-			1.00			
3.5-4.0	1.09	(0.84	-	1.42)	0.68	(0.22	-	2.11)	1.07	(0.87	-	1.30)
3.0-3.5	0.82	(0.63	-	1.07)	0.57	(0.18	-	1.79)	0.97	(0.80	-	1.19)
≤3.0	0.69	(0.49	-	0.95)	0.24	(0.06	-	0.88)	0.72	(0.56	-	0.91)
**Allowance**												
Q1 (Low)	1.00	-			1.00	-			1.00			
Q2	0.75	(0.60	-	0.94)	0.84	(0.40	-	1.76)	0.76	(0.64	-	0.89)
Q3	0.55	(0.43	-	0.71)	0.49	(0.20	-	1.21)	0.53	(0.45	-	0.64)
Q4 (High)	0.51	(0.39	-	0.66)	0.20	(0.08	-	0.49)	0.44	(0.36	-	0.53)
**Living Status**												
Family Home	1.00	-			1.00	-			1.00			
Living Alone/Flatting	0.63	(0.51	-	0.79)	0.66	(0.32	-	1.36)	0.64	(0.55	-	0.75)
College Dorm	0.80	(0.64	-	1.00)	1.14	(0.49	-	2.61)	0.73	(0.61	-	0.86)
**Smoking Status**												
Current Smoker	0.50	(0.39	-	0.63)	1.03	(0.47	-	2.25)	0.54	(0.46	-	0.64)
Past Smoker	0.36	(0.24	-	0.54)	0.71	(0.21	-	2.41)	0.56	(0.40	-	0.77)
Non-Smoker	1.00	-			1.00	-			1.00			
**Underage Drinking**												
Yes	0.78	(0.65	-	0.94)	0.23	(0.12	-	0.46)	0.43	(0.37	-	0.49)
No	1.00	-			1.00	-			1.00			
**Number of sororities/clubs**												
None	1.00	-			1.00	-			1.00			
One	0.89	(0.73	-	1.07)	0.56	(0.30	-	1.06)	1.05	(0.91	-	1.20)
Two or more	0.63	(0.47	-	0.85)	0.72	(0.29	-	1.80)	0.84	(0.67	-	1.06)

R-Square = current flushers: 0.21114; former flushers: 0.3509; never flushers: 0.2134; Goodness of fit [26] = 9007.1; former flushers: 987.8; never flushers: 18020.4.
